# Single-cell analysis reveals the lncRNA-MEG3/miRNA-133a-3p/PRRT2 axis regulates skeletal muscle regeneration and myogenesis

**DOI:** 10.1016/j.gendis.2022.04.012

**Published:** 2022-05-05

**Authors:** Yilong Yao, Zishuai Wang, Yun Chen, Lei Liu, Liyuan Wang, Guoqiang Yi, Yalan Yang, Dazhi Wang, Kui Li, Zhonglin Tang

**Affiliations:** a Shenzhen Branch, Guangdong Laboratory of Lingnan Modern Agriculture, Agricultural Genomics Institute at Shenzhen, Chinese Academy of Agricultural Sciences, Shenzhen, Guangdong 518000, China; b Kunpeng Institute of Modern Agriculture at Foshan, Foshan, Guangdong 528226, China; c Center for Regenerative Medicine University of South Florida Health Heart Institute, Morsani College of Medicine, University of South Florida, FL 33602, USA; d Genome Analysis Laboratory of the Ministry of Agriculture and Rural Affairs, Agricultural Genomics Institute at Shenzhen, Chinese Academy of Agricultural Sciences, Shenzhen, Guangdong 518000, China; e GuangXi Engineering Centre for Resource Development of Bama Xiang Pig, Bama, Guangxi 547500, China; f State Key Laboratory of Animal Nutrition, Institute of Animal Science, Chinese Academy of Agricultural Sciences, Beijing 100193, China

Skeletal muscle is the largest motor and metabolic organ of the body, which has a robust capacity for regeneration following injury or disease. Delayed regeneration after skeletal muscle injury reduces muscle contractility and leads to dysfunction of innervation. Therefore, identifying the regulation components in skeletal muscle regeneration and determining their molecular mechanisms are important to discover novel therapeutic markers for muscular diseases. Long non-coding RNA (LncRNA) has been implicated in skeletal muscle regeneration. Recent developed single-cell RNA sequencing (scRNA-seq) provides a higher resolution of cellular differences than bulk RNA-seq. Here, we re-analyzed single-cell transcriptomes data of skeletal muscle regeneration and identified lncRNA maternally expressed gene 3 (*lncRNA-MEG3*) was highly expressed in muscle satellite cells (MuSCs). Further study showed that *lncRNA-MEG3* regulates skeletal muscle regeneration via sponging miR-133a-3p to regulate proline-rich transmembrane protein 2 (*PRRT2*) expression level. These results suggested that *lncRNA-MEG3* might be a potential target for skeletal muscle diseases.

To identify critical lncRNAs associated with muscle regeneration at single-cell level, we re-analyzed scRNA-seq dataset generated by De Micheli (Fig. S1A).^1^ By combining the scRNA-seq atlas (Fig. S1B) and histology of regenerating muscle (Fig. S1C), we discovered that day 5 post-injury was a critical time point for muscle regeneration. Subsequently, we investigated the expression of lncRNAs on day 5 following injury. 764 lncRNAs were expressed in at least one cell (Fig. S1D) and six lncRNAs highly expressed in MuSCs, myofibroblasts and fibro/adipogenic progenitors (FAPs) with *lncRNA-MEG3* exhibited the greatest abundance in MuSCs (Fig. 1A). For confirmation, we re-analyzed other scRNA-seq datasets for skeletal muscle^2^^,^^3^ and obtained consistent results (Fig. S1E). During muscle regeneration, *lncRNA-MEG3* expression was induced on day 3 and peaked on day 5 post injury (Fig. 1B). Interestingly, based on re-analysis of scRNA-seq data published by He et al,^4^ we found that *lncRNA-MEG3* was upregulated in MuSCs transitioned from quiescent to differentiated during embryonic development (Fig. S1F, G). Real time quantitative PCR (RT-qPCR) results showed that *lncRNA-MEG3* was abundantly expressed in skeletal muscle at postnatal day 0 and downregulated from postnatal day 0 to day 65 (Fig. S1H, I). Together, these findings suggested that *lncRNA-MEG3* might be a potential regulator of skeletal muscle regeneration.

**Figure 1:**
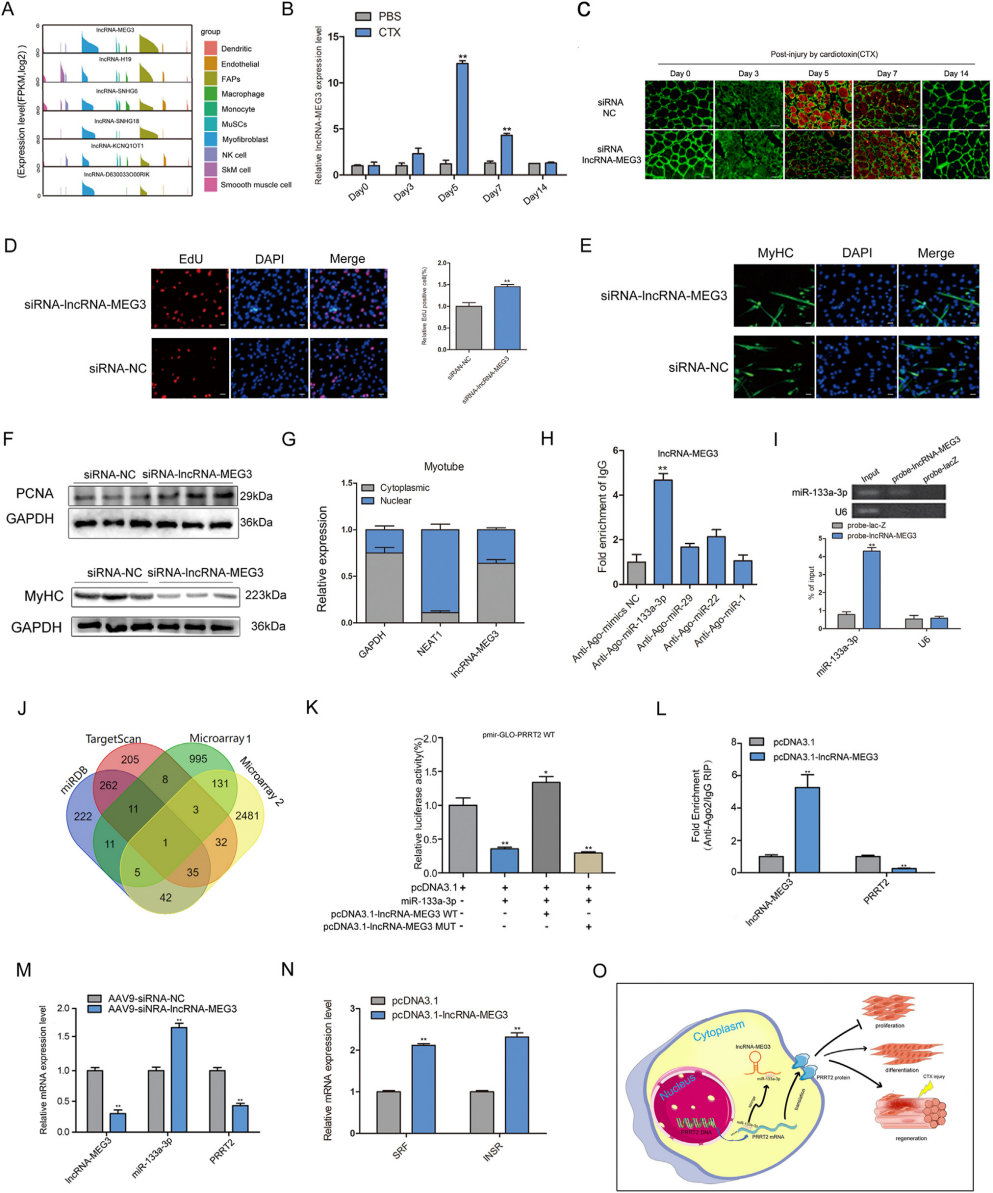
*LncRNA-MEG3* promotes skeletal muscle differentiation and regeneration via acting as a ceRNA against miR-133a-3p, which further upregulated *PRRT2* expression. **(A)** Expression level of 6 lncRNAs in all cell types at 5 days after injury. **(B)** RT-qPCR analysis of *lncRNA-MEG3* expression in damaged TA muscle on different days (*n =* 3). **(C)** Co-immunofluorescence staining for laminin and MyHC-embryo; scale bar, 50 μm. Red color indicates the newly formed muscle fiber, green color represents all of the muscle fiber. **(D)** EdU-staining analysis for cell proliferation in the siRNA-NC and siRNA-*lncRNA-MEG3* groups, nuclei were stained with DAPI; scale bar, 50 μm (*n =* 3). **(E)** Immunofluorescence staining analysis of differentiated primary myoblasts transfected with siRNA-NC and siRNA-*lncRNA-MEG3* groups and then cultured in DM for 4 days. Nuclei were stained with DAPI. Scale bar, 50 μm. **(F)** Western blotting analysis of *PCNA* and *MyHC* expression levels in primary myoblasts after transfection with siRNA-NC or siRN*A-lncRNA-MEG3* (*n =* 3). **(G)** Nuclear-cytoplasmic separation test of *lncRNA-MEG3*, *NEAT1*, and *GAPDH* in C2C12 myoblasts differentiated in DM for 3 days. (*GAPDH* as cytoplasmic marker; *NEAT1* as nuclear marker) (*n* = 3). **(H)** RIP-qPCR assay was performed to verify the interaction between miRNAs and *lncRNA-MEG3* after overexpression miR-1, miR-22, miR-29 and miR-133a-3p (*n* = 3). **(I)** RAP-qPCR assay for the amount of miR-133a-3p in TA muscle. MiR-133a-3p and *U6* expression levels were detected by PCR (up panel). RT-qPCR results showed miR-133a-3p enrichment by *lncRNA-MEG3* prob and lacz prob (NC) (down panel) (*n* = 3). **(J)** Venn diagram of overlapping target genes. The target genes were predicted with Targetscan, miRDB, Microarray assay of C2C12 transfected with siRNA-*lncRNA-MEG3* (Microarray 1), and Microarray assay of skeletal muscles from postnatal day 0 and day 65 (Microarray 2). **(K)** Determination of luciferase activities in different treatment groups (*n* = 3). HEK293T cells were co-transfected with pcDNA3.1, miR-133a-3p, pcDNA3.1-*lncRNA-MEG3* WT and pcDNA3.1-*lncRNA-MEG3* MUT plasmid. **(L)** RIP-qPCR analysis of the enrichment of *lncRNA-MEG3* and *PRRT2* in control pcDNA3.1 and pcDNA3.1-*lncRNA-MEG3* groups (*n* = 3). **(M)** RT-qPCR analysis of miRNA-133a-3p, *PRRT2,* and *lncRNA-MEG3* expression level after *lncRNA-MEG3* knockdown *in vivo* (*n* = 3). **(N)** RT-qPCR analysis of *SRF* and *INSR* mRNA expression levels in C2C12 myoblasts after *lncRNA-MEG3* overexpression (*n* = 3). **(O)** The mechanism graph of the *lncRNA-MEG3* regulatory network. Data are expressed as mean values ± SEM, and a paired two-tailed Student's *t*-test was used to analyze the statistical significance between two groups. ∗∗*P* < 0.01, and ∗*P* < 0.05.

Next, a regeneration model by tibialis anterior (TA) muscle injection was used to determine the role of *lncRNA-MEG3* during skeletal muscle regeneration *in vivo* (Fig. S2A–C). In the groups of *lncRNA-MEG3* knockdown, the peak of newly formed myofibers appeared at 7 days post injury, which was 2 days delayed compared to the control group (Fig. 1C; Fig. S2D–H). Single-cell transcriptome analysis showed that immune cells were increased in the *lncRNA-MEG3* knock-down group at 5 days post injury (Fig. S2I–K) further indicating that *lncRNA-MEG3* knockdown delayed the kinetics of skeletal muscle regeneration.

Given skeletal muscle regeneration relies on the skeletal muscle satellite cell myogenesis, we used functional gain and loss to study the effects of *lncRNA-MEG3* on primary myoblasts (Fig. S3). The cell-counting-kit-8 (CCK-8) assay, 5-ethynyl-2′-deoxyuridine (EdU)-staining, immunofluorescence (IF), RT-qPCR and Western blotting results showed that *lncRNA-MEG3* knockdown significantly improved the C2C12 proliferation and inhibited differentiation, while cells treated with *lncRNA-MEG3* overexpression vector showed the opposite phenomenon (Fig. 1D, F; Fig. S4). Moreover, the roles of *lncRNA-MEG3* in C2C12 were consistent with that in primary myoblasts (Fig. S5).

The localization of lncRNA in the cell is assumed to be a marker for determining the regulatory mechanisms of lncRNA.^5^ We found *lncRNA-MEG3* is mainly expressed in cytoplasm of C2C12 myotube (Fig. 1G). So, we speculated that the *lncRNA-MEG3* might act as ceRNA in myogenesis. Microarray profiling of lncRNAs and miRNAs expression in TA muscle from day 0 to day 65 were performed (Fig. S6A–D; Table S1, S2) to identify miRNAs interact with *lncRNA-MEG3*. Accordingly, miR-133a-3p was selected from four myomiRs using RNA immunoprecipitation (RIP)-qPCR (Fig. 1H). This was further confirmed by RNA antisense purification (RAP)-qPCR (Fig. 1I). Subsequently, the luciferase activity of pmirGLO-*lncRNA-MEG3*-WT was declined after miR-133a-3p over-expression, which was rescued by increasing the concentration of *lncRNA-MEG3* (Fig. S6E–G). Furthermore, the expression level changes of miR-133a-3p was opposite with *lncRNA-MEG3* during skeletal muscle development, C2C12 myoblasts differentiation, and regeneration (Fig. S6H–J). To further determine the regulatory relationship, we subsequently conducted the rescue experiments. The results confirmed that *lncRNA-MEG3* repressed the effect of miR-133a-3p on C2C12 proliferation and differentiation (Fig. S7, S8).

To identify genes that were involved in the ceRNA network, we performed microarray profiling of the mRNA transcriptome in *lncRNA-MEG3* knockdown C2C12 and skeletal muscle at postnatal day 0 and day 65 (Fig. S9A–C and Table S3–5). Interestingly, only *PRRT2* was identified after bioinformatic analysis (Fig. 1J). In addition, the expression levels of *PRRT2* and *lncRNA-MEG3* were positively correlated during skeletal muscle development and C2C12 differentiation, and the *PRRT2* mRNA expression level was upregulated after *lncRNA-MEG3* overexpression (Fig. S9D–H). Further experiments showed that *PRRT2* is a target gene of miR-133a-3p and it could inhibit C2C12 proliferation and promote differentiation (Fig. S9I–L, S10, 11).

Subsequently, we further confirmed *lncRNA-MEG3/*miR-133a-3p*/PRRT2 ceRNA* regulatory network. The dual-luciferase assay results showed that the activity of pmir-GLO-*PRRT2* WT vector was reduced after miR-133a-3p overexpression, which was reversed by the addition of pcDNA3.1-*lncRNA-MEG3* WT but not pcDNA3.1-*lncRNA-MEG3* MUT plasmid (Fig. 1K). RIP-qPCR assay showed that overexpression of *lncRNA-MEG3* caused a significant decrease in the enrichment of *PRRT2* level (Fig. 1L). In addition, knockdown of *lncRNA-MEG3* increased the expression of miR-133a-3p and downregulated the expression of *PRRT2* in TA muscle (Fig. 1M). The expression of *SRF* and *INSR* as miR-133a-3p targets were regulated by overexpression or silencing *lncRNA-MEG3* expression level (Fig. 1N; Fig. S12A). Moreover, the effects of miR-133a-3p on C2C12 myoblasts were significantly reversed by *lncRNA-MEG3* overexpression vector (Fig. S12B–E). Collectively, these results showed that *lncRNA-MEG3* serves as a ceRNA for miR-133a-3p to regulate *PRRT2* expression level during skeletal muscle regeneration (Fig. 1O).

## Ethics declaration

All animal procedures were performed according to the guidelines of Institutional Animal Care and Use Committee of Agricultural Genomics Institute at Shenzhen, Chinese Academy of Agricultural Sciences.

## Author contributions

Zhonglin Tang designed the study and revised the manuscript. Zishuai Wang and Yilong Yao wrote the manuscript. Yilong Yao, Zishuai Wang and Yun Chen performed molecular and cellular experiments; Liyuan Wang and Yun Chen performed the single cell assay; Lei Liu and Zishuai Wang performed single cell RNA-seq analysis; Yun Chen helped with cell culture, cell transfection, qPCR, vector construction, EdU-staining and western blotting. Guoqiang Yi, Yalan Yang, and Dazhi Wang helped revise the manuscript.

## Conflict of interests

The author(s) declare that they have no conflict of interest.

## Funding

This work was supported by The National Natural Science Foundation of China (No. 31830090); The Basic and Applied Basic Research Foundation of Guangdong province, China (No. 2019B1515120059); The Agricultural Science and Technology Innovation Program, China (No. CAAS-ZDRW202006).

## Consent for publication

All authors have agreed to publish this manuscript.
