# Supplementary material for: Single-cell analysis reveals the lncRNA-MEG3/miRNA-133a-3p/PRRT2 axis regulates skeletal muscle regeneration and myogenesis
Source: Genes Dis. 2022 May 5;10(2):359–62. doi: 10.1016/j.gendis.2022.04.012 (PMC10201586; doi:10.1016/j.gendis.2022.04.012)
Supplement: Multimedia component 2 [file mmc2.docx]

**Supplementary Materials and Methods**

**Animal studies**

The C57BL/6 mice (Hufukang, Beijing) were sacrificed according to the welfare requirements for experimental animals (12/12 h light/dark cycle and free access to food and water).

**Mice regeneration model**

A mice regeneration model was generated by injecting cardiotoxin into the tibialis anterior (TA) muscles of sixty 8-week-old male C57BL/6 mice (Hufukang, Beijing). After injection, the muscles were harvested at various times (0.5, 1, 2, 3, 4, 5, 6, 7, 14, 21 or 28 day), whereas un-injected TA muscles were used as control.

**Cell culture**

Mice C2C12 myoblasts and HEK-293T cells were obtained from Peking Union Medical College Hospital and cultured in Dulbecco’s Modified Eagle’s Medium (DMEM, Gibco, USA) supplemented with 10% fetal bovine serum and 1% penicillin-streptomycin (PS, Thermo Scientific, USA) at 37 °C in a 5% CO_2_ incubator. To induce myogenic differentiation, the culture medium of C2C12 myoblasts was replaced with DMEM supplemented with 2% horse serum (Gibco, USA) and 1% PS at 80% confluence.

**Isolation and differentiation of mice primary myoblast**

Primary myoblasts were isolated from 2- to 5-day-old C57BL/6 mice as described by Ge et al., 2011 [^1^](#_ENREF_1) and cultured in DMEM/F12 -Dulbecco's Modified Eagle Medium (DMEM/F12, Gibco, USA) supplemented with 10% fetal bovine serum and 1% penicillin-streptomycin (PS, Thermo Scientific, USA) at 37 °C in a 5% CO_2_ incubator. Differentiation was induced at 50–70% cell density using differentiation medium as described above for 4 days, and the medium was changed daily.

**Primers and oligonucleotides**

Primers were designed using Primer Premier5. The *PRRT2* siRNA, *lncRNA-MEG3* siRNA, siRNA-NC, all miRNA mimics, mimics NC, miRNA inhibitor, and inhibitor NC were obtained from GenePharma (Shanghai, China). All sequences are provided in Table S6.

**Nuclear and Cytoplasmic RNA Fractionation**

Cytoplasmic and nuclear RNAs were extracted from C2C12 cells at differentiated periods with protocols described previously [^2^](#_ENREF_2). RNA from the cytoplasmic and nuclear fractions were extracted with RNAiso reagent (TaKaRa, Otsu, Japan). *NEAT1*, *GAPDH* and *lncRNA-MEG3* were detected by RT-qPCR. *NEAT1* is a known nuclear lncRNA, and *GAPDH* is a cytoplasmic-enriched gene. The primers are listed in Table S7.

**5-Ethynyl-2’-deoxyuridine (EdU) assay**

Myoblast cells were seeded in 12-well plates. At 50% confluence, cells were transfected with overexpression plasmid, siRNA, miRNA mimics or control. After transfection for 48 h, myoblasts were exposed to 50 μM EdU (RiboBio, China) for 2 h at 37°C. Subsequently, cells were fixed in 4% paraformaldehyde for 30 min, neutralized using 2 mg/mL glycine solution, and permeabilized with 0.5% Triton X-100. A solution containing EdU (Apollo Reaction Cocktail; RiboBio, China) was added, and the cells were incubated at room temperature for 30 min. The nuclear stain Hoechst 33342 was then added, and the incubation period was extended for another 30 min. A fluorescence microscope (DMi8; Leica, German) was used to capture three randomly selected fields to visualize the number of EdU-stained cells.

**RNA extraction and Real-time quantitative polymerase chain reaction (RT-qPCR)**

Total RNA was extracted using TRIzol™ Reagent (Invitrogen, Shanghai, China). For lncRNA and mRNA, HiScript III 1st Strand cDNA Synthesis kit (+gDNA wiper) (R312-01, Vazyme, Nanjing, China) was used to generate cDNA following the manufacturer’s instructions. For miRNA the reverse transcription was performed with miRNA 1st Strand cDNA Synthesis Kit (MR101-01, Vazyme, Nanjing, China).

RT-qPCR was performed on a 7500 FAST Real‐Time PCR System (Applied Biosystems) according to the Taq Pro Universal SYBR qPCR Master Mix instructions (Q712-02, Vazyme, Nanjing, China). The reaction conditions were 95°C for 10 s, 40 cycles of 5 s at 95°C, and 30 s at 60°C. The relative gene expression levels were analyzed using the 2^-△△CT^ method. As for the endogenous reference genes, mouse *GAPDH* and *U6* were used to normalize gene expression levels. The primers are listed in Table S7.

**Western blotting**

After transfection for 48 h, cells were lysed in RIPA buffer (Thermo Scientific 89901, Massachusetts, USA) supplemented with a protease inhibitor cocktail (Roche 04693132001, Basel Switzerland) and a phosphatase inhibitor (Roche 5892791001, Basel, Switzerland). The extracts were boiled with 4× SDS loading buffer (CWBIO; Beijing, China) at 100°C for 20 min, after which western blotting was performed as previously described [^3^](#_ENREF_3). Antibodies against MyHC (developmental myosin 1:1000, DSHB MF20, Iowa, USA), PRRT2 (1:1000, Bioss bs-19430R, Beijing, China), PCNA (1:1000, Abcam ab18197, Cambridge, UK), and GAPDH (Abcam ab9482, Cambridge, UK) were obtained from DSHB (Iowa, USA), Bioss (Beijing, China), and Abcam (Cambridge, UK), respectively. Densitometric analysis of the protein bands was performed using the ImageJ software (NIH, Bethesda, MD, USA).

**Plasmid construction**

The pcDNA3.1 vector (Invitrogen, Carlsbad, CA, USA) covering *PRRT2* coding sequences (pcDNA3.1-PRRT2) and full-length transcript sequences of *lncRNA-MEG3* (pcDNA3.1-lncRNA-MEG3) were structured to overexpress indicated genes, with the empty vector (pcDNA3.1) as the negative control. The target gene fragment was amplified by ApexHF HS DNA Polymerase FS Master Mix (AG12202, accurate biotechnology) using forward and reverse primers containing *BamHI* and *XhoI* sites. PCR products were inserted into the pcDNA3.1.

The WT and MUT seed sequences of miR-133a-3p in the *lncRNA-MEG3* gene were ligated into pcDNA3.1 (pcDNA3.1-lncRNA-MEG3 WT and pcDNA3.1-lncRNA-MEG3 MUT). RT-qPCR analysis of miR-133a-3p expression levels in C2C12 cells was performed after transfection with 50 nM pcDNA3.1-lncRNA-MEG3 WT, pcDNA3.1-lncRNA-MEG3 MUT overexpressing vectors, and pcDNA3.1 (control) in growth medium. Blank, pcDNA3.1, and siRNA-NC were used as control.

The putative miR-133a-3p target-binding sequence in wild-type *lncRNA-MEG3* (pmir-GLO-lncRNA-MEG3 WT) and the mutant (pmir-GLO-lncRNA-MEG3 MUT) of binding sequence for miR-133a-3p were synthesized and cloned downstream of luciferase gene in pmir-GLO dual-luciferase miRNA target expression vectors (Promega, Madison, WI, USA). This vector is based on Promega dual-luciferase technology, with firefly luciferase (luc2) used as the primary reporter to monitor mRNA regulation and Renilla luciferase (hRluc-neo) acting as a control reporter for normalization and selection. To determine whether miR-133a-3p directly targets *PRRT2*, we constructed wild-type *PRRT2* reporter plasmids (pmir-GLO-PRRT2 WT) and mutant plasmids by changing binding sequences of *PRRT2* (pmir-GLO-PRRT2 Mut). The primers are listed in Table S7.

**Targeted gene prediction**

The miRNA binding sites were predicted using Targetscan (<http://www.targetscan.org/mamm_31/>), miRDB (<http://mirdb.org/>), and RNAhybrid (<http://bibiserv.techfak.xn--unibielefeld-i09f.de/rnahybrid/>).

**Dual-luciferase reporter assay**

HEK293T cells were grown to 75–80% confluence in 12 well plates and subsequently co-transfected with 50 nM pmir-GLO-lncRNA-MEG3 WT, pmir-GLO-lncRNA-MEG3 Mut, pmir-GLO-PRRT2 WT, pmir-GLO-PRRT2 Mut vector and 50 nM NC, miR-133a-3p mimics using Lipofectamine™ 3000 (Invitrogen) according to the manufacturer’s instructions. Cells were harvested after 24 h, and luciferase activity was evaluated using a dual-luciferase assay system (Promega).

**Cell counting kit-8 proliferation assay**

C2C12 myoblasts and primary myoblasts were seeded in a 96-wells plate with 5,000 cells/well. Cells were harvested after 0 h, 24 h, 48 h, and 72 h. According to the manufacturer's instructions, the proliferation of myoblasts was measured using the Cell Counting Kit-8 (CCK-8) (Beyotime C0038, Beijing, China). Each sample's optical density (OD) was measured using a microplate reader (Tecanspark, Switzerland) at a wavelength of 450 nm, and a proliferation curve was constructed.

**Immunofluorescence analysis**

Skeletal muscle was frozen in liquid nitrogen and sliced into 5 μm-thick sections. Cells were cultured in 6-well plates and fixed with paraformaldehyde. Sections and cells were treated with 0.5% Triton, blocked with goat serum for 1 h, and incubated with anti-embryonic MyHC (1:500, Elabscience E-AB-22021, Wuhan, China), anti-Laminin (1:500, Abcam, ab11575, Cambridge, UK), anti-development MyHC (1:500, DSHB MF20, Shanghai, China) antibodies anti-PAX7 (1:500, Abcam, ab187339, Cambridge, UK) and anti-MyOD (1:500, Affinity Biosciences, AF7733, American) for 2 h, respectively. Subsequently, cells and sections were incubated with a goat anti-mice secondary antibody. Finally, DAPI (1:1000, Invitrogen D3571, Shanghai, China) was added, and the cells were observed using a Leica DMI3000 B microscope (Leica).

**RNA immunoprecipitation (RIP) based on Ago2**

C2C12 myoblasts were transfected with vectors or microRNA mimics. After 48 h, cells were used to perform RIP experiments with an anti-Ago2 antibody (Abcam ab186733, Cambridge, UK) and the Magna RIP™ RNA-Binding Protein Immunoprecipitation Kit (Millipore, Bedford, MA, USA) according to the manufacturer’s instructions. RNAs were isolated from the immunoprecipitation products and quantitated using a Nano-Photometer spectrophotometer (Implen, Munich, Germany). RT-qPCR was performed to examine the expression levels of *lncRNA-MEG3* and *PRRT2*.

**RNA antisense Purification (RAP)**

The RAP experiment was performed according to the kit instructions (BersinBio^TM^, Bes5103-1, Guangzhou, China). A weight of 0.4 g mice TA skeletal muscle was cut into pieces and cross-linked with 1% paraformaldehyde for 20 min. A mixture of tissue and 1% paraformaldehyde was added to 1.375 M glycine and centrifuged. Cells were collected after homogenate precipitation and lysed in lysis buffer containing protease and RNA inhibitors, DNase salt, EDTA, EGTA, and DTT. Cell lysates were divided into treatment, control, and input groups. *LncRNA-MEG3*-probes (5’-GCAGCACTCCAGTTCACTAGCAGATGAACACGAGCACAGATGTA C-3’) and hybridization buffer were added to the treatment group. LacZ-probes (5’-CAAACGGCGGATTGACCGTAATGGGATAGGTCACGTTGGTGTAGATGGGCGCATCGTAAC-3’) and hybridization buffer were added to the control group. Subsequently, magnetic beads were used to adsorb RNA from the treatment and control groups. RNA (including the input sample) was extracted and the expression of miR-133a-3p was detected after reverse transcription.

**Hematoxylin-eosin (H&E) staining**

Skeletal muscle tissue samples were fixed in 4% paraformaldehyde for 24–36 h, after which paraffin-embedded tissues were sliced into 5 μm-thick sections. Subsequently, the sections were dehydrated, stained with H&E, dehydrated again, cleared, and mounted with neutral resin [^4^](#_ENREF_4).

**Preparation of Adeno-Associated Virus (AAV) Serotype 9 (AAV9) and muscle Injections**

Adeno-Associated Virus Serotype 9 (AAV9) carried-siRNA-NC (AAV9-siRNA-NC) control vector, and AAV9 carried the siRNA targeting the *lncRNA-MEG3* vector were supplied by Hanbio (China). The sequence of siRNA (5’-GCTACGGGTTTGCACACTTTT-3’) was subjected to generate AAV9-lncRNA-MEG3-siRNA. The expression of either *lncRNA-MEG3* siRNA or NC siRNA was driven by *U6* promoter, whereas *EGFP* expression was driven by the [cytomegalovirus](https://www.sciencedirect.com/topics/biochemistry-genetics-and-molecular-biology/cytomegalovirus) (CMV) promoter in the AAV9 vector.

For the AAV9 virus administration experiment, we used sixty 8-weeks-old male C57BL/6 mice (Hufukang, Beijing). AAV9-siRNA-lncRNA-MEG3 were injected into both ends of one side of the TA muscles of each mouse at a dosage of 1×10¹³ vg/mL. A total of 100 μL of viral preparation was injected into each muscle. AAV9-siRNA-NC was injected into the left leg, and AAV9-siRNA-lncRNA-MEG3 were injected into the right leg. At 28 days after injection of AAV9, 100 μL (10 Μm) CTX (Cardiotoxin, Sigma-Aldrich) was injected, and the TA muscles were collected on days 0, 1, 3, 5, 7, and 14.

**Single-cell library preparation and RNA-sequencing**

Five days after CTX injection, the siRNA-lncRNA-MEG3 and siRNA-NC TA muscles were isolated and digested with collagenase I (800 U/mL) and Dispase II (11 U/mL) for 30 minutes. The digestive residue was screened and filtered using a 40-µm cell strainer, after which it was washed twice with DPBS (+2% fetal bovine serum). Next, library construction and single-cell RNA-sequencing were performed according to the instructions of the Chromium Next GEM Single Cell 3 Reagent Kits v3.1 kit. Briefly, the cells were diluted using the Chromium Single Cell A Chip to yield about 6,000 single-cell transcriptomes with < 5% doublet rate. Following the library preparation, the libraries were sequenced in multiplex (*n* = 2 per sequencing run) on the NextSeq 500 sequencer (Illumina, San Diego, CA) to generate between 200 and 250 million reads per library and an average of 30,000 reads per single cell.

**Analysis of scRNA-seq data**

Sequencing reads were processed with Cell Ranger version 3.0.1 (10X Genomics, Pleasanton, CA) using the mouse reference transcriptome mm10. Downstream analysis was carried out with R version 3.6.2 (2019-12-12) based on the gene expression matrix. Quality control, filtering, data clustering, data visualization, and differential expression analysis were carried out using Seurat version 2.3.4 R package with some custom modifications to the standard pipeline. Genes expressed in less than 3 cells and cells expressed < 200 genes were removed. In addition, we removed any single-cell with > 20% mitochondrial genes. The gene expression matrix was subjected to PCA analysis, with the first 15 principal components used for clustering and visualization. Unsupervised shared nearest neighbor (SNN) clustering with a resolution of 0.4 was conducted, and visualization was carried out using Uniform Manifold Approximation and Projection (UMAP). Differential expression analysis was performed with Seurat's 'FindAllMarkers' function, which employs a likelihood ratio test that assumes the data has a negative binomial distribution and only considers genes with > log2 (0.25) fold-change and expressed in at least 25% of the cells in the cluster. For pesudotime analysis, we subsetted the cells labeled as MuSCs from the dataset and across all time points and samples. And then cells were organized in pseudotime using R package Monocle[^5^](#_ENREF_5) version 3 using default parameters.

**Microarray analysis**

Gastrocnemius muscle 0–65 days after birth and C2C12 myoblasts transfected with siRNA-*lncRNA-MGE3* or siRNA-NC were collected. An ArraryStar mice lncRNA microarray was used to determine the expression profiles of lncRNAs and protein-coding transcripts. Scanned images were imported into Agilent Feature Extraction software (version 11.0.1.1) for grid alignment and data extraction. Quantile normalization and subsequent data processing were performed using the GeneSpring GX v12.1 software package (Agilent Technologies). After quantile normalization of the raw data, lncRNAs and mRNAs for which at least 3 out of 6 samples had flags of “Present” or “Marginal” (“All Targets Value”) were chosen for further data analysis. Differentially expressed lncRNAs and mRNAs with statistical significance between the two groups were identified through *p* -value/FDR filtering (|fold change| ≥ 2.0 and *p* ≤ 0.05).

An Agilent mice miRNA microarray was used to determine the expression profiles of the miRNAs. Scanned images were imported into the GenePix Pro 6.0 software (Axon) for grid alignment and data extraction. Replicated miRNAs were averaged, and miRNAs with intensities ≥ 30 in all samples were chosen for the calculation of the normalization factor. Expression data were normalized using median normalization, after which significantly differentially expressed miRNAs between the two groups were identified according to fold change and *p*-value (|fold change| ≥ 2.0 and *p* ≤ 0.05).

**Image J software analysis**

We used Image J to quantify the average EdU and fluorescence intensity according to the “Mean = IntDen / Area (Mean: Mean gray value; IntDen: Integrated Density)” formula. The operation process of Image J is as follows: (1) Image-Color-Split Channels; (2) Image-Adjust-Threshold; (3) Image-Adjust-Auto Threshold; (4) Analyze-Set Measurements. The average gray value and threshold limit need to be selected in this step; (5) Analyze-Measure.

**Data availability**

The data generated in the present study can be downloaded in raw and processed forms from the NCBI Gene Expression Omnibus under accession number GSE155070 (microarray) and PRJNA648252 (scRNA-seq).

**Statistical analysis**

Results are presented as the mean ± SEM. GraphPad Prism v. 7.0 (GraphPad Software, La Jolla, CA, USA) was used for statistical analysis. All differences between groups were analyzed by one-way analysis of variance (ANOVA) and the Student’s *t*-test. A significance threshold of *p* <0.05 was used to determine statistically significant results.

**Reference**

1. Ge Y, Sun Y, Chen J. IGF-II is regulated by microRNA-125b in skeletal myogenesis. *J Cell Biol.* 2011;192(1):69-81.

2. Yu X, Zhang Y, Li T, et al. Long non-coding RNA Linc-RAM enhances myogenic differentiation by interacting with MyoD. *Nat Commun.* 2017;8:14016.

3. Zhang J, Ying ZZ, Tang ZL, Long LQ, Li K. MicroRNA-148a promotes myogenic differentiation by targeting the ROCK1 gene. *Journal of Biological Chemistry.* 2012;287(25):21093.

4. Ying Y, Xu H, Yao M, Qin Z. Protective effect of hydrogen-saturated saline on acute lung injury induced by oleic acid in rats. *J Orthop Surg Res.* 2017;12(1):134.

5. Cole T, Davide C, Jonna G, et al. The dynamics and regulators of cell fate decisions are revealed by pseudotemporal ordering of single cells. *Nat biotechnol*. 2014;32(4):381-386.
