# Supplementary material for: Single-cell analysis reveals the lncRNA-MEG3/miRNA-133a-3p/PRRT2 axis regulates skeletal muscle regeneration and myogenesis
Source: Genes Dis. 2022 May 5;10(2):359–62. doi: 10.1016/j.gendis.2022.04.012 (PMC10201586; doi:10.1016/j.gendis.2022.04.012)
Supplement: Multimedia component 3 [file mmc3.docx]

**
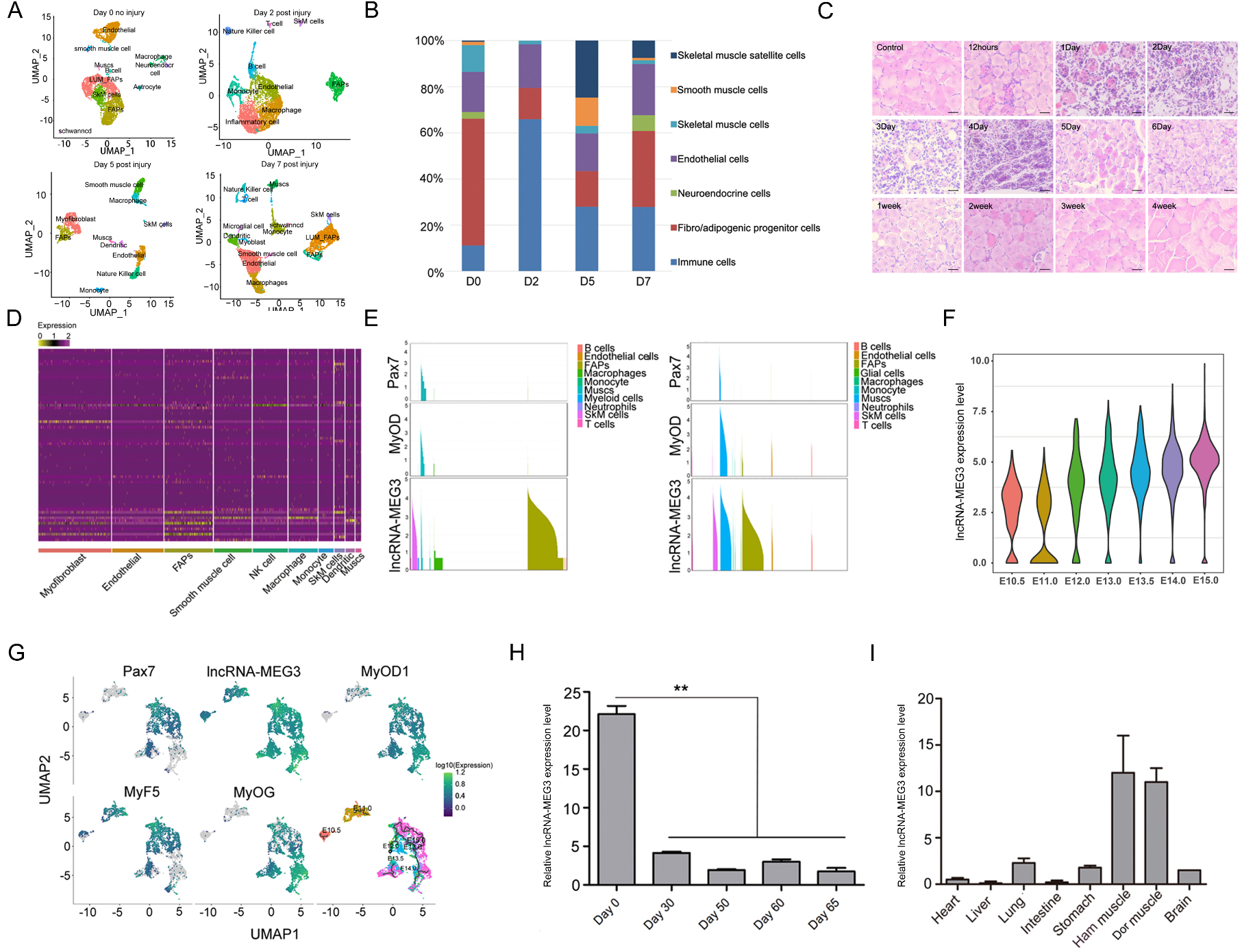
Supplementary Information**

**Figure S1.** Single-cell RNA-seq analysis revealed the participation of *lncRNA-MEG3* in mice skeletal muscle regeneration. **(A)** Single cells from the mouse muscle regeneration experiments split by time-points post-injury on UMAP plots and colored by cell type. Nature killer cells (NK cells); skeletal muscle cells (skm cell); fibro/adipogenic progenitors (FAPs). **(B)** Compositional dynamics of cell types throughout the regeneration time course. **(C)** H&E staining of TA muscle after cardiotoxin (CTX) injection; scale bar, 50 μm. **(D)** The expression patterns of 764 lncRNAs at day 5 after injury in the single-cell RNA-seq data. **(E)** ScRNA-seq analysis *lncRNA-MEG3* expression in different cell clusters. Left panel data from Oprescu et al[^1^](#_ENREF_1), right panel data from Gordani et al[^2^](#_ENREF_2). *PAX7* and *MyoD* were used to mark MuSCs. **(F)** Violin plots presenting *lncRNA-MEG3* expression changes during embryonic development. **(G)** *PAX7*, *MyOD*, *MyOG,* and *lncRNA-MEG3* expression levels within the myogenic cells during embryonic development. **(H)** RT-qPCR analysis of *lncRNA-MEG3* expression in tibialis anterior (TA) muscle at day 0, day 30, day 50, day 60, and day 65 after birth (*n* = 3). **(I)** RT-qPCR analysis of *lncRNA-MEG3* expression in eight different tissues of mice at postnatal day 0 (*n* = 3). Data are expressed as mean values ± SEM, and a paired two-tailed Student’s *t*-test was used to analyze the statistical significance between two groups. ∗∗ *p* < 0.01 and ∗ *p* < 0.05.


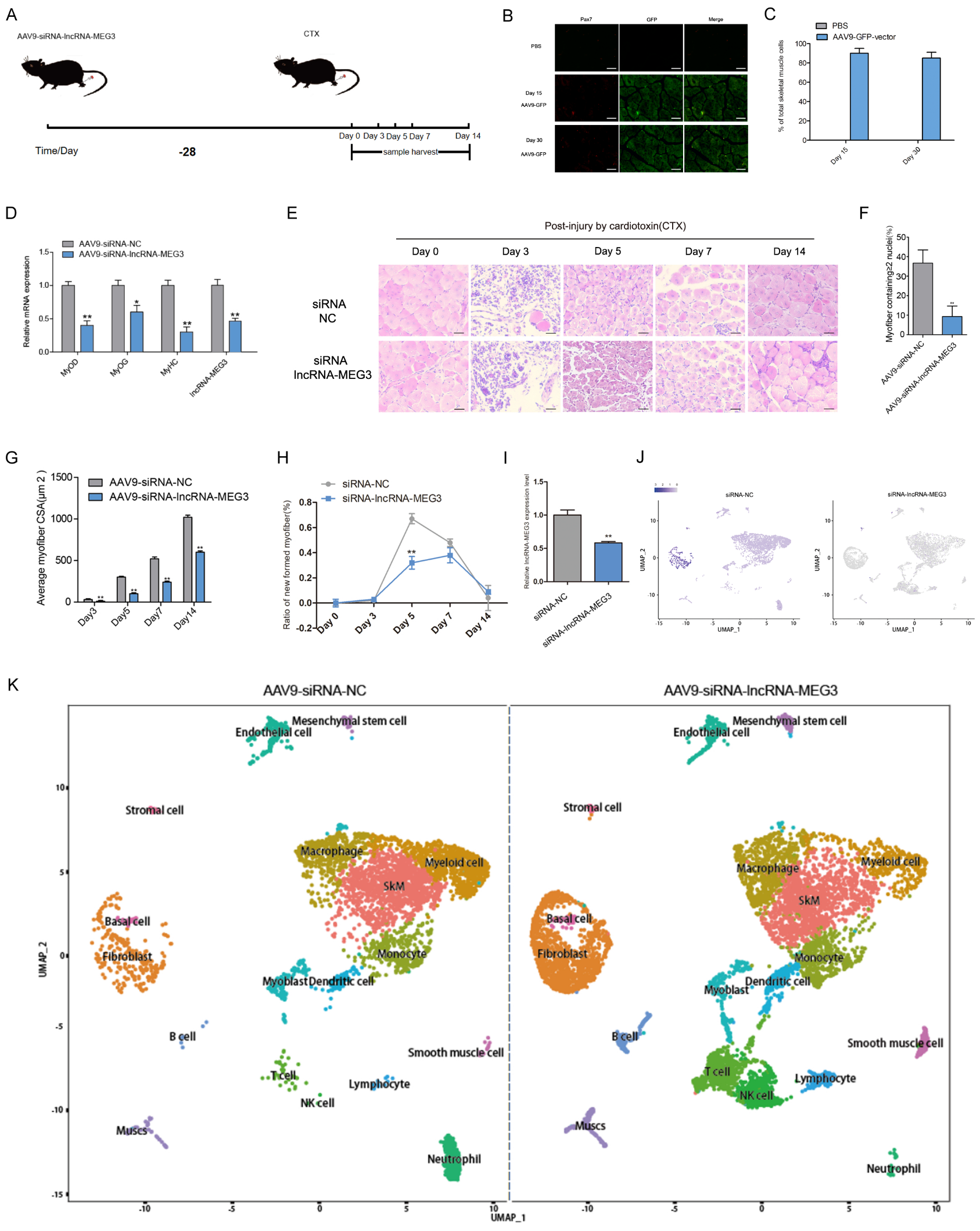


**Figure S2.** *LncRNA-MEG3* is critical for skeletal muscle regeneration. **(A)** Schematic diagram of Adeno-Associated Virus Serotype 9 (AAV9) injection and muscle regeneration experiments. AAV9-siRNA-NC (control) was injected into the left leg of mice, and AAV9-siRNA-lncRNA-MEG3 was injected into the right leg. **(B)** The infection efficiency of AAV9 in mice TA muscle was detected at day 15 and day 30; scale bar, 50μm. *Pax7* is Red, *GFP* is green (*n =* 3). **(C)** Quantification of *GFP* positive cells of muscle fiber (*n =* 3). Quantification was conducted with Image Pro Plus. **(D)** RT-qPCR analysis of *lncRNA-MEG3* knockdown efficiency and *M*y*OD*, *MyOG* and *MyHC* mRNA expression at 28 days after infection by AAV9-siRNA-NC and AAV9-siRNA-*lncRNA-MEG3* (*n* = 3). **(E)** Comparison of H&E staining for the regeneration of TA muscle following injury; scale bar, 50 μm (*n* = 3). **(F)** Quantitation of regenerating myofibers with more than two nuclei at day 5 using ImageJ software (*n* = 3). **(G)** Cross-section area of regenerating fibers on different days using Image Pro Plus (*n* = 3). **(H)** The ratio of new formed myofibers on different days using Image Pro Plus. More than 200 fibers were counted in each group (*n* = 3). **(I)** RT-qPCR analysis of *lncRNA-MEG3* knockdown efficiency in the injured TA muscle at 5 days (*n* = 3). **(J)** Single-cell RNA-seq analysis of *lncRNA-MEG3* expression level in different cell clusters. **(K)** Uniform Manifold Approximation and Projection (UMAP) plots showing single cells injected with CTX colored by cell type and split by treatment: knock-down *lncRNA-MEG3* (AAV-siRNA-lncRNA-MEG3) and control (AAV-siRNA-NC). Data are expressed as mean values ± SEM, and a paired two-tailed Student’s *t*-test was used to analyze the statistical significance between two groups. ∗∗ *p* < 0.01 and ∗ *p* < 0.05.

**
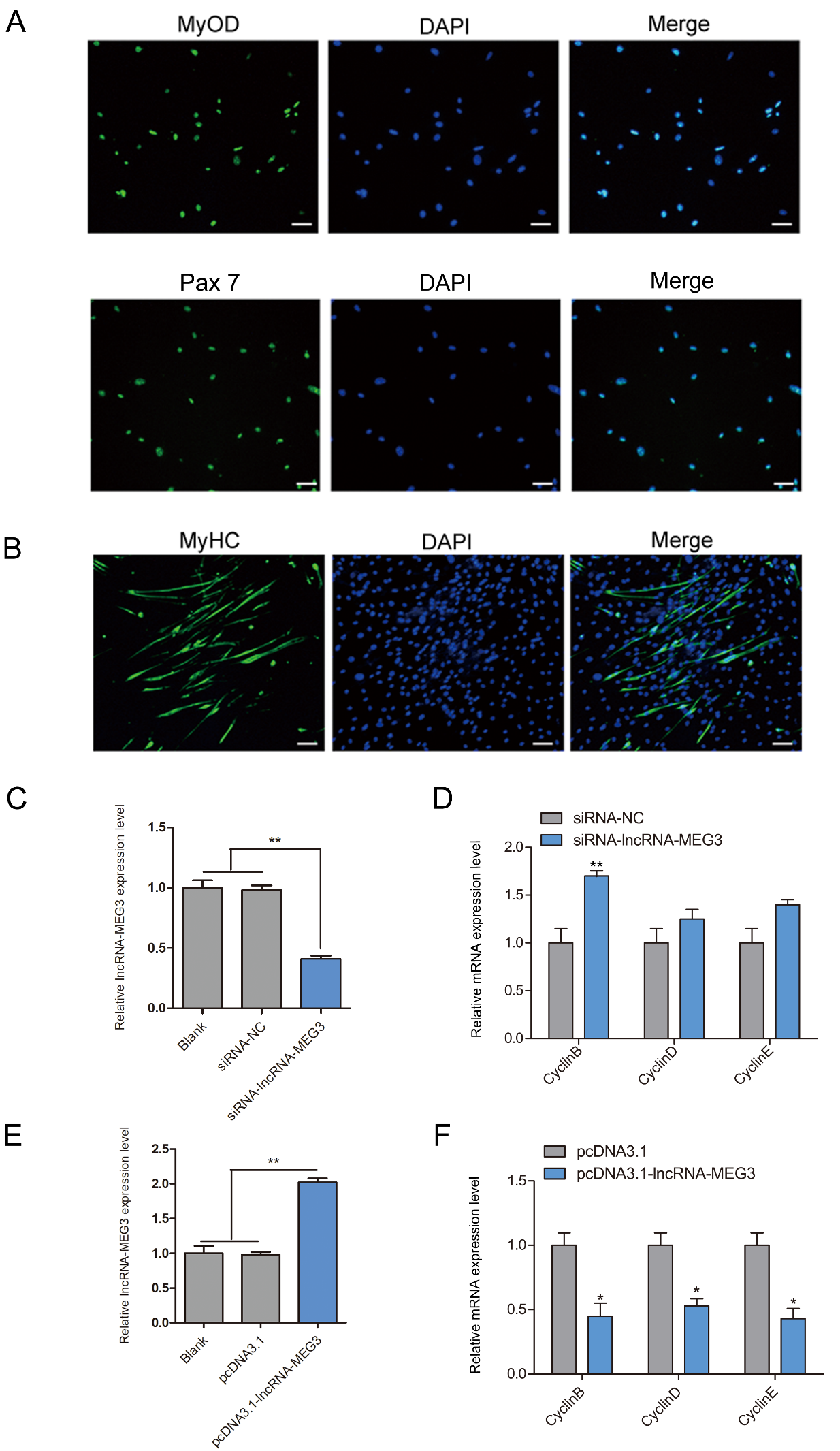
**

**Figure S3.** *LncRNA-MEG3* regulates cell cycle gene expression in mice primary myoblast. **(A)** Immunofluorescence staining analysis of *MyOD* and *PAX7* in mice skeletal muscle primary myoblasts (*n =* 3); scale bar 50 μm. **(B)** Immunofluorescence staining analysis of *MyHC* in primary myoblasts differentiated cells for 4 days (*n =* 3); scale bar 100 μm. Nuclei were stained with DAPI. **(C)** RT-qPCR analysis of *lncRNA-MEG3* knockdown efficiency (*n =* 3). Blank, pcDNA3.1, and siRNA-NC as control. **(D)** RT-qPCR analysis cell cycle marker gene (*CyclinB*, *CyclinD* and *CyclinE*) expression in siRNA-NC and siRNA-lncRNA-MEG3 groups (*n =* 3). **(E)** RT-qPCR analysis of *lncRNA-MEG3* overexpression efficiency (*n =* 3). Blank, pcDNA3.1, and siRNA-NC as control. **(F)** RT-qPCR analysis cell cycle marker gene (*CyclinB*, *CyclinD* and *CyclinE*) expression in control pcDNA3.1 and pcDNA3.1-lncRNA-MEG3 groups (*n =* 3). Data are expressed as mean values ± SEM, and a paired two-tailed Student’s *t*-test was used to analyze the statistical significance between two groups. ** *p* <0.01, and * *p* < 0.05.


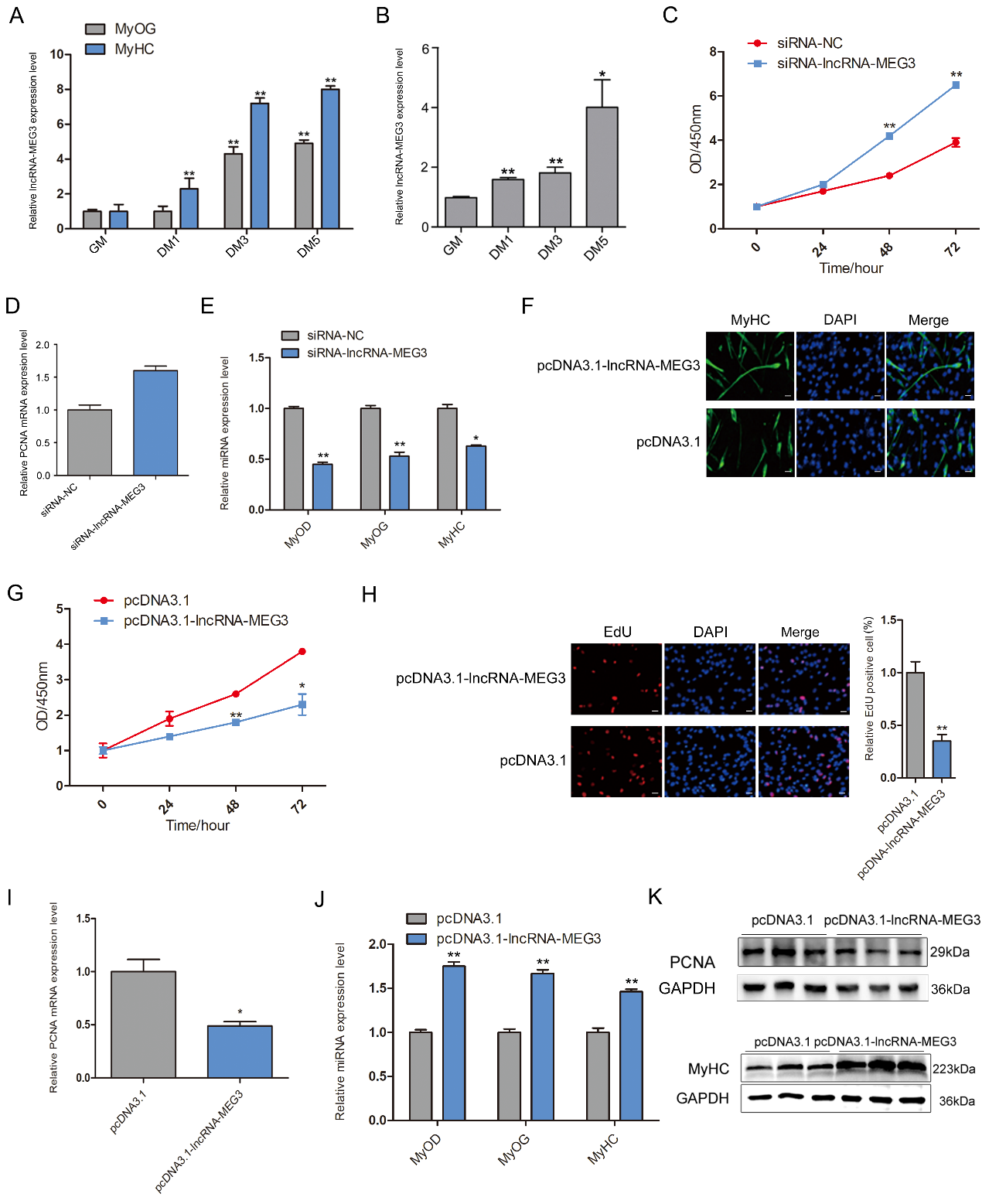


**Figure S4.** *LncRNA-MEG3* promotes mice primary myoblast differentiation. **(A)** RT-qPCR analysis of *MyOG* and *MyHC* mRNA expression levels in C2C12 myoblasts differentiated for 4 days (*n =* 3). **(B)** RT-qPCR analysis of *lncRNA-MEG3* expression in differentiated C2C12 myoblasts (*n* = 3). **(C)** CCK-8 assay analysis for cell proliferation in primary myoblasts after transfection with siRNA-NC or siRNA-lncRNA-MEG3 (*n =* 3). **(D)** RT-qPCR analysis of *PCNA* expression level in primary myoblasts after transfection with siRNA-NC or siRNA-lncRNA-MEG3 (*n =* 3). **(E)** RT-qPCR analysis of *MyOD*, [*MyOG*](https://www.sciencedirect.com/topics/biochemistry-genetics-and-molecular-biology/myod) and *MyHC* in primary myoblasts differentiated for 4 days after transfection with siRNA-NC or siRNA-lncRNA-MEG3 (*n =* 3). **(F)** Immunofluorescence staining analysis of primary myoblasts differentiation for 4 days in control pcDNA3.1 and pcDNA3.1-lncRNA-MEG3 groups. Nuclei were stained with DAPI. Scale bar, 50 μm (*n =* 3). **(G)** CCK-8 assay analysis for cell proliferation in primary myoblasts after transfection with control pcDNA3.1 or pcDNA3.1-lncRNA-MEG3 (*n =* 3). **(H)** EdU-staining analysis for cell proliferation in control pcDNA3.1 and pcDNA3.1-lncRNA-MEG3 groups, nuclei were stained with DAPI. Scale bar, 50 μm (*n =* 3). **(I)** RT-qPCR analysis of *PCNA* expression level in primary myoblasts after transfection with control pcDNA3.1 or pcDNA3.1-lncRNA-MEG3 (*n =* 3). **(J)** RT-qPCR analysis of *MyOD*, [*MyOG*](https://www.sciencedirect.com/topics/biochemistry-genetics-and-molecular-biology/myod) and *MyHC* in primary myoblasts differentiated for 4 days after transfection with control pcDNA3.1 or pcDNA3.1-lncRNA-MEG3 (*n =* 3). **(K)** Western blotting analysis of *PCNA* and *MyHC* protein expression level in primary myoblasts after *lncRNA-MEG3* overexpression or knockdown (*n =* 3). Data are expressed as mean values ± SEM, and a paired two-tailed Student's *t*-test was used to
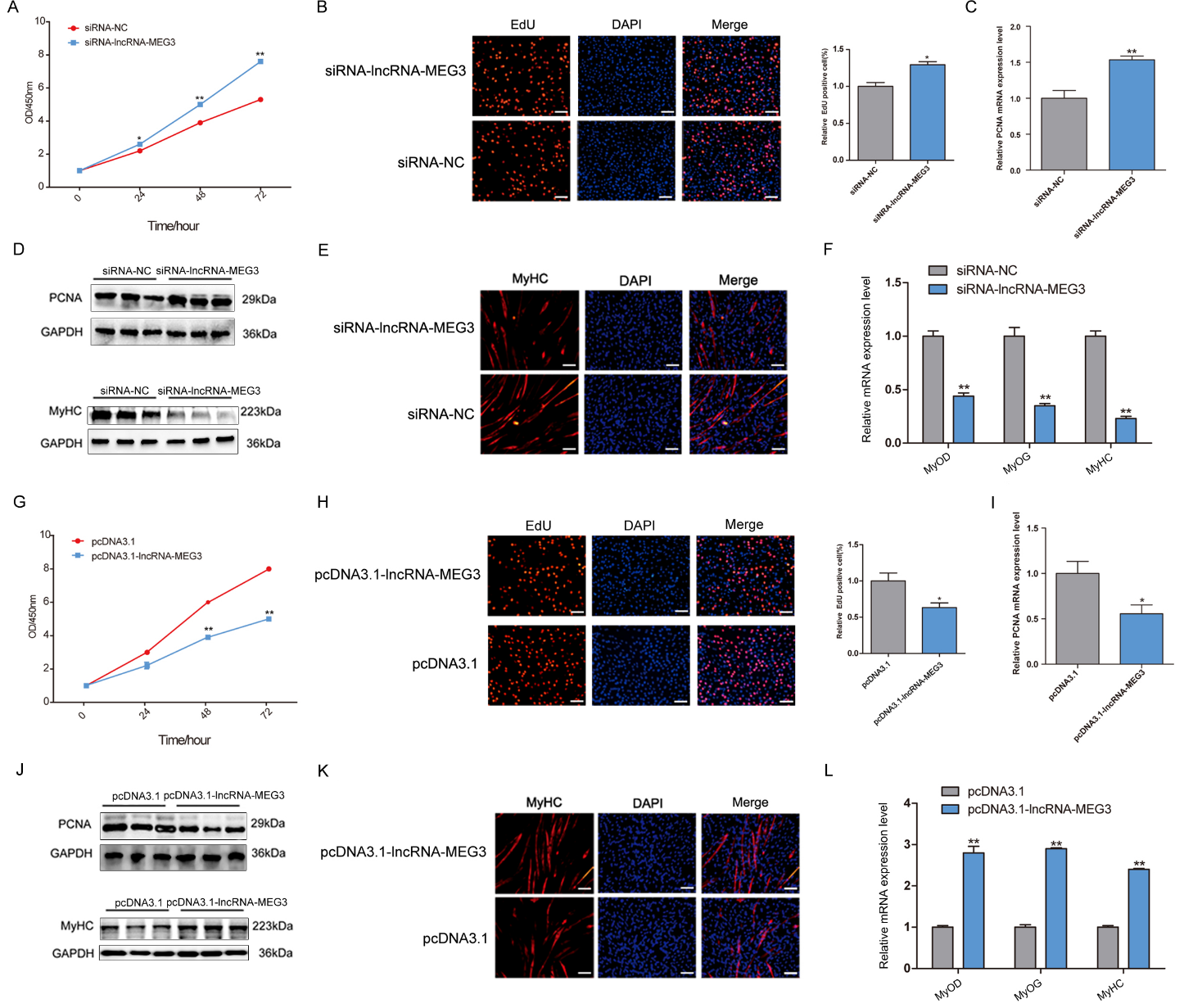
analyze the statistical significance between two groups. ** *p* < 0.01 and * *p* <0.05.

**Figure S5.** *LncRNA-MEG3* regulates the proliferation and differentiation of C2C12 myoblasts. **(A)** CCK-8 assay analysis of cell proliferation after transfection with siRNA-NC or siRNA-lncRNA-MEG3 (*n* = 3). **(B)** EdU-staining analysis for cell proliferation in the siRNA-NC and siRNA-lncRNA-MEG3 groups, nuclei were stained with 4’,6-Diamidino-2’-phenylindole (DAPI); scale bar, 50 μm (*n* = 3). **(C)** RT-qPCR analysis of *PCNA* mRNA expression level in C2C12 myoblasts after transfection with siRNA-NC or siRNA-lncRNA-MEG3 (*n* = 3). **(D)** Western blotting analysis of *PCNA* and *MyHC* protein expression level in C2C12 myoblasts after lncRNA-MEG3 knockdown (*n* = 3). **(E)** Immunofluorescence staining analysis of C2C12 differentiation for 4 days in siRNA-NC and siRNA-lncRNA-MEG3 groups. Nuclei were stained with DAPI. Scale bar, 50 μm (*n* = 3). **(F)** RT-qPCR analysis of *MyOD*, [*MyOG*](https://www.sciencedirect.com/topics/biochemistry-genetics-and-molecular-biology/myod), and *MyHC* in C2C12 myoblasts differentiated for 4 days after transfection with siRNA-NC or siRNA-lncRNA-MEG3 (*n* = 3). **(G)** CCK-8 assay analysis cell proliferation after transfection with control pcDNA3.1 or pcDNA3.1-lncRNA-MEG3 (*n* = 3). **(H)** EdU-staining analysis for cell proliferation in control pcDNA3.1 and pcDNA3.1-lncRNA-MEG3 groups, nuclei were stained with (DAPI); scale bar, 50 μm (*n* = 3). **(I)** RT-qPCR analysis of *PCNA* mRNA expression level after transfection with control pcDNA3.1 or pcDNA3.1-lncRNA-MEG3 (*n* = 3). **(J)** Western blotting analysis of *PCNA* and *MyHC* protein expression level after transfection with control pcDNA3.1 or pcDNA3.1-lncRNA-MEG3 (*n* = 3). **(K)** Immunofluorescence staining analysis of C2C12 differentiation for 4 days in control pcDNA3.1 and pcDNA3.1-lncRNA-MEG3 groups. Nuclei were stained with DAPI. Scale bar, 50 μm (*n* = 3). **(L)** RT-qPCR analysis of *MyOD*, [*MyOG*](https://www.sciencedirect.com/topics/biochemistry-genetics-and-molecular-biology/myod), and *MyHC* in C2C12 cells differentiated for 4 days after transfection with control pcDNA3.1 or pcDNA3.1-lncRNA-MEG3 (*n* = 3). Data are expressed as mean values ± SEM, and a paired two-tailed Student’s *t*-test was used to analyze the statistical significance between two groups. ∗∗ *p* < 0.01 and ∗ *p* < 0.05.


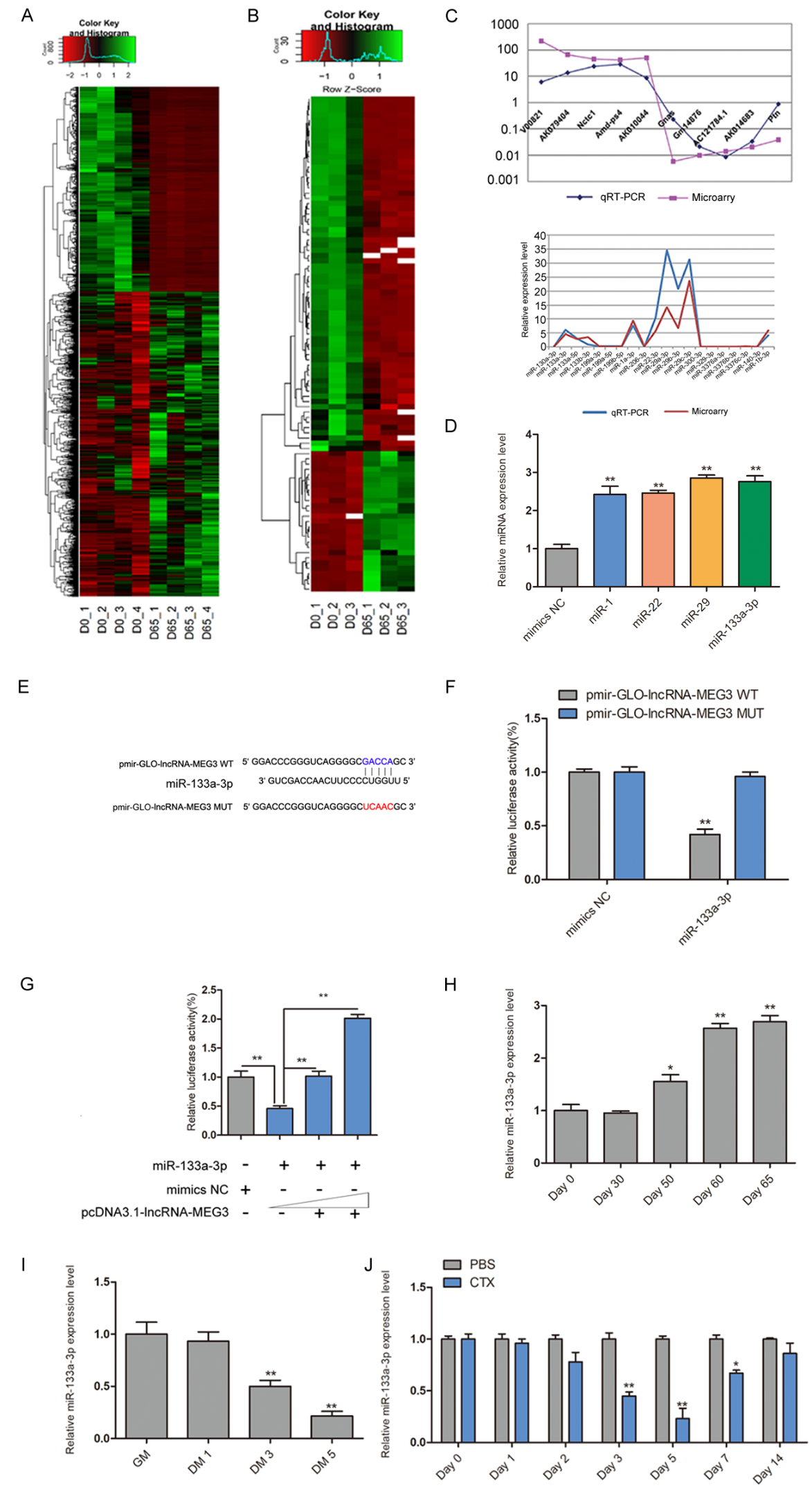


**Figure S6.** MiR-133a-3p sponged by *lncRNA-MEG3*. **(A, B)** Heatmaps showing the expression profiles of 3350 lncRNAs (left panels) and 95 miRNAs (right panel) with different signals at postnatal day 0 and day 65. Green indicates low intensity, black indicates medium intensity, and red indicates strong intensity. **(C)** RT-qPCR was performed to validate the lncRNAs (upper panel) and miRNAs (down panel) randomly selected from the microarray data (*n =* 3). **(D)** RT-qPCR analysis of overexpression efficiency in C2C12 myoblasts transfected with miR-1, miR-22, miR-29 or miR-133a-3p mimics (*n =* 3). **(E)** Dual-luciferase reporter vector was constructed, including miR-133a-3p wild and mutation binding sites of *lncRNA-MEG3*. Blue is wild-type binding sites. Red is mutation-type binding sites. **(F)** Dual-luciferase activity was performed after transfection with miRNA mimics and mimics NC (*n* = 3). **(J)** Dual-luciferase activity was detected by co-transfection miR-133a-3p and different concentration (0 ug, 1.6 ug and 3.2 ug) of *lncRNA-MEG3* overexpression plasmid (*n* = 3). RT-qPCR analysis miR-133a-3p expression in skeletal muscle development **(H)** differentiated C2C12 myoblasts **(I)** and injured TA muscle **(J)** (*n* = 3). Data are expressed as mean values ± SEM, and a paired two-tailed Student’s *t*-test was used to analyze the statistical significance between two groups. ** *p* < 0.01, and * *p* < 0.05.


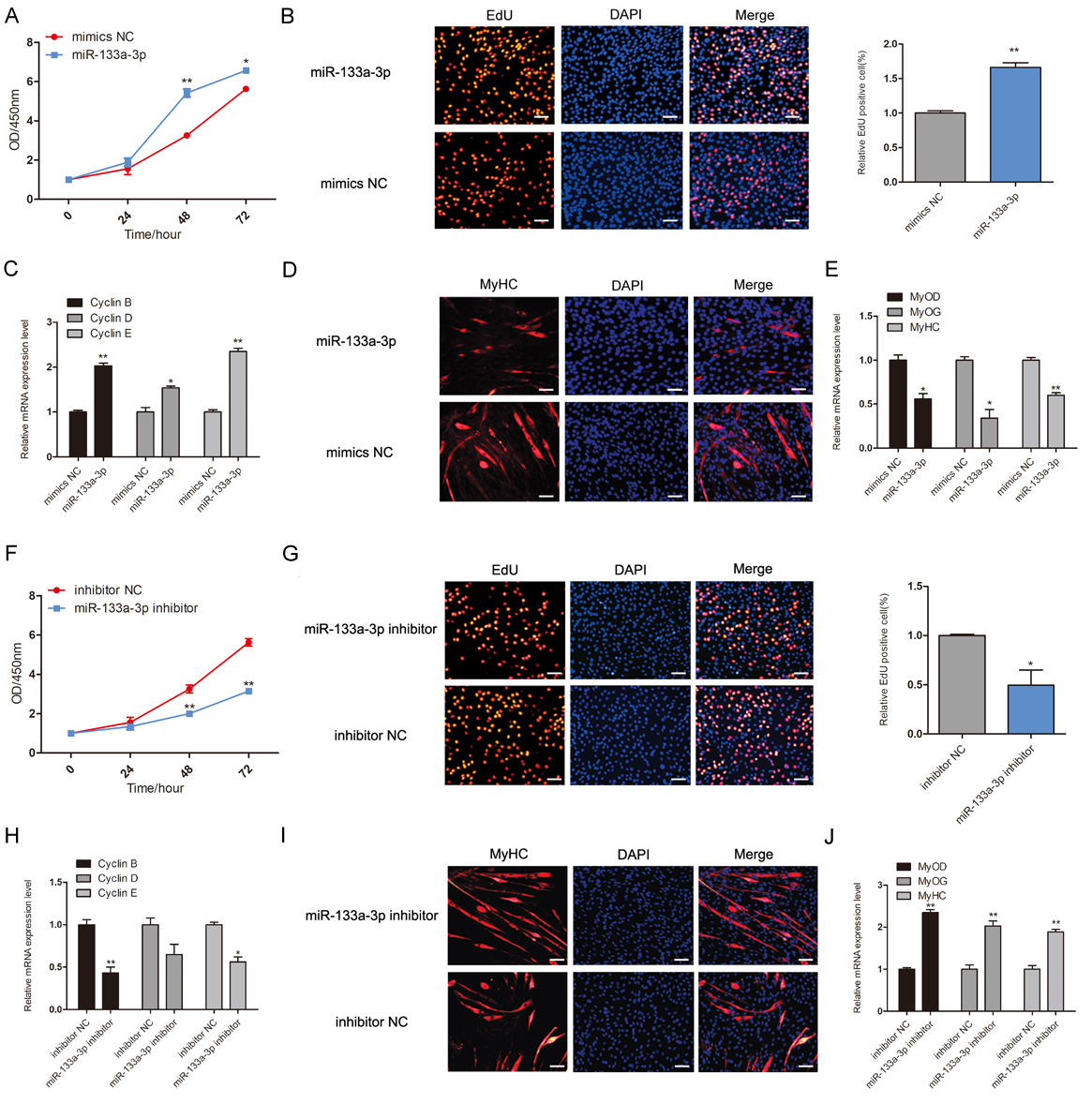


**Figure S7.** C2C12 myoblasts proliferation and differentiation were regulated by miR-133a-3p. **(A-E)** C2C12 myoblasts proliferation and differentiation were determined after transfection with mimics NC or miR-133a-3p mimics. **(A-B)** CCK-8 assay and EdU-staining analysis of cell proliferation in different treatment groups, nuclei were stained with DAPI; scale bar, 50 μm (*n =* 3). **(C)** RT-qPCR analysis of cell cycle marker (*CyclinB*, *CyclinD* and *CyclinE*) expression in different treatment groups (*n =* 3). **(D)** Immunofluorescence staining analysis of C2C12 differentiation in different treatment groups. Nuclei were stained with DAPI; scale bar, 50 μm (*n =* 3). **(E)** RT-qPCR analysis of *MyOD*, *MyOG,* and *MyHC* mRNA expression in different treatment groups (*n =* 3). **(F-G)** C2C12 myoblasts proliferation was determined by CCK-8 assay and EdU-staining after the miR-133a-3p knockdown. **(H)** Cell cycle marker genes expression was determined by RT-qPCR after the miR-133a-3p knockdown. **(I)** Immunofluorescence staining analysis of C2C12 differentiation after the miR-133a-3p knockdown. Nuclei were stained with DAPI; scale bar, 50 μm (*n =* 3). **(J)** RT-qPCR analysis of *MyOD*, *MyOG* and *MyHC* mRNA expression after miR-133a-3p knockdown (*n =* 3). Data are expressed as mean values ± SEM, and a paired two-tailed Student’s *t*-test was used to analyze the statistical significance between two groups. **
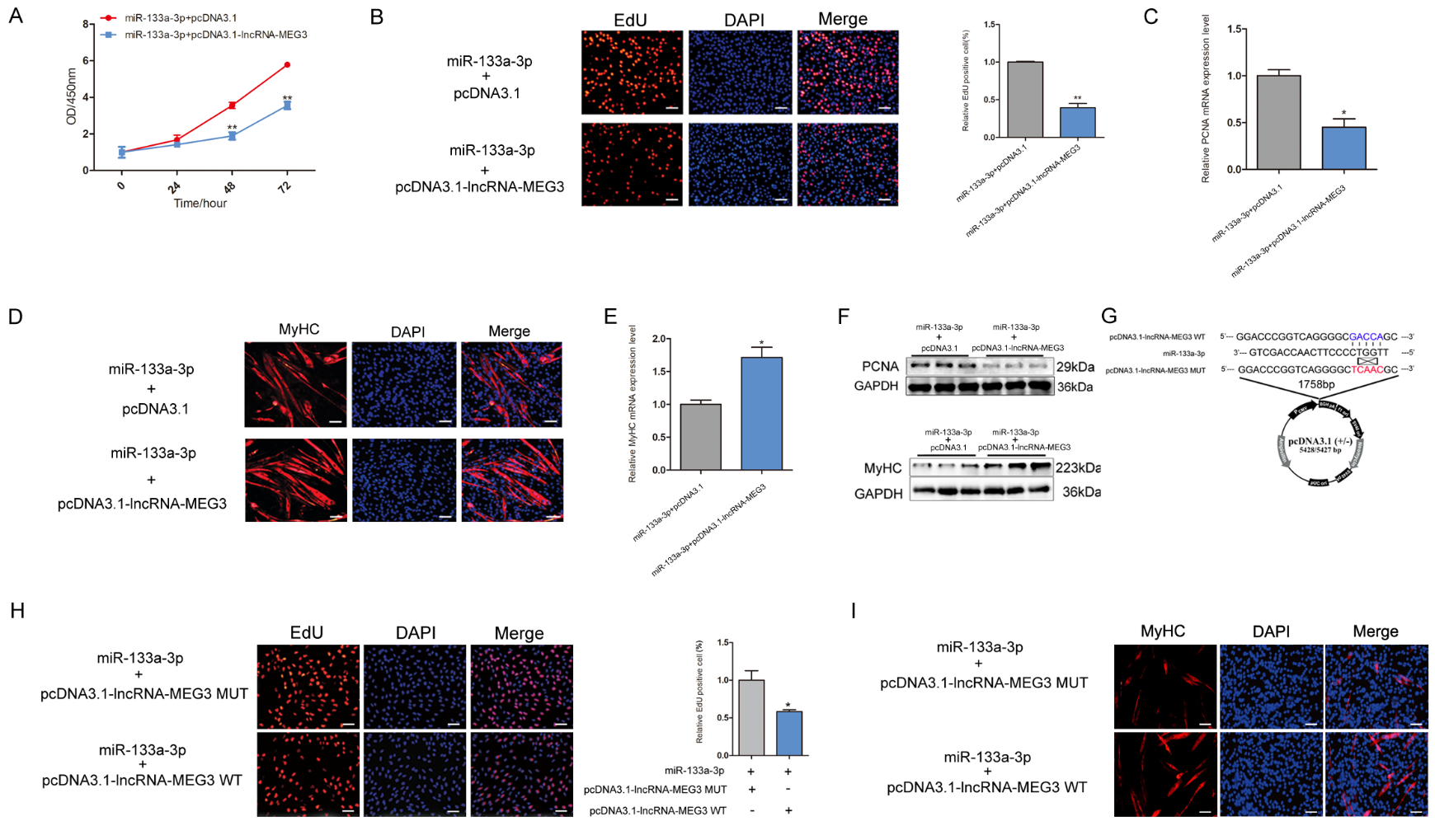
*p* <0.01, and * *p* < 0.05.

**Figure S8.** Validation of miR-133a-3p as a target binding miRNA of *lncRNA-MEG3* in C2C12 myoblasts. **(A-****C)** [C2C12](https://www.sciencedirect.com/topics/biochemistry-genetics-and-molecular-biology/c2c12) myoblasts were co-transfected with miR-133a-3p+pcDNA3.1 and miR-133a-3p+pcDNA3.1-lncRNA-MEG3. **(A, B)** Cell proliferation was determined by CCK-8 assay and EdU-staining. Nuclei were stained with DAPI; scale bar, 50 μm (*n* = 3). **(C)** RT-qPCR analysis of *PCNA* expression (*n* = 3). **(D, E)** [C2C12](https://www.sciencedirect.com/topics/biochemistry-genetics-and-molecular-biology/c2c12) myoblasts were co-transfected with miR-133a-3p+pcDNA3.1 and miR-133a-3p+pcDNA3.1-lncRNA-MEG3 and then differentiated for four days. **(D)** Immunofluorescence staining analysis of C2C12 differentiation in different treatment groups. Nuclei were stained with DAPI; scale bar, 50 μm (*n* = 3). **(E)** RT-qPCR analysis of *MyHC* mRNA expression level in different treatment groups (*n* = 3). **(F)** Western blotting analysis of *PCNA* and *MyHC* protein expression level after transfection with miR-133a-3p+pcDNA3.1 and miR-133a-3p+pcDNA3.1-lncRNA-MEG3 (*n* = 3). **(G)** Construction of vectors including wild-type (pcDNA3.1-lncRNA-MEG3 WT) and mutant (pcDNA3.1-lncRNA-MEG3 MUT) sequences of *lncRNA-MEG3* cDNA. **(H)** EdU-staining and Immunofluorescence staining **(I)** analysis for C2C12 differentiation and proliferation after transfection with miR-133a-3p+pcDNA3.1*-lncRNA-MEG3* MUT or miR-133a-3p+pcDNA3.1-lncRNA-MEG3 WT, nuclei were stained with DAPI; scale bar, 50 μm (*n* = 3). Data are expressed as mean values ± SEM, and a paired two-tailed Student’s *t*-test was used to analyze the statistical significance between two groups. ∗∗ *p* < 0.01 and ∗ *p* < 0.05.


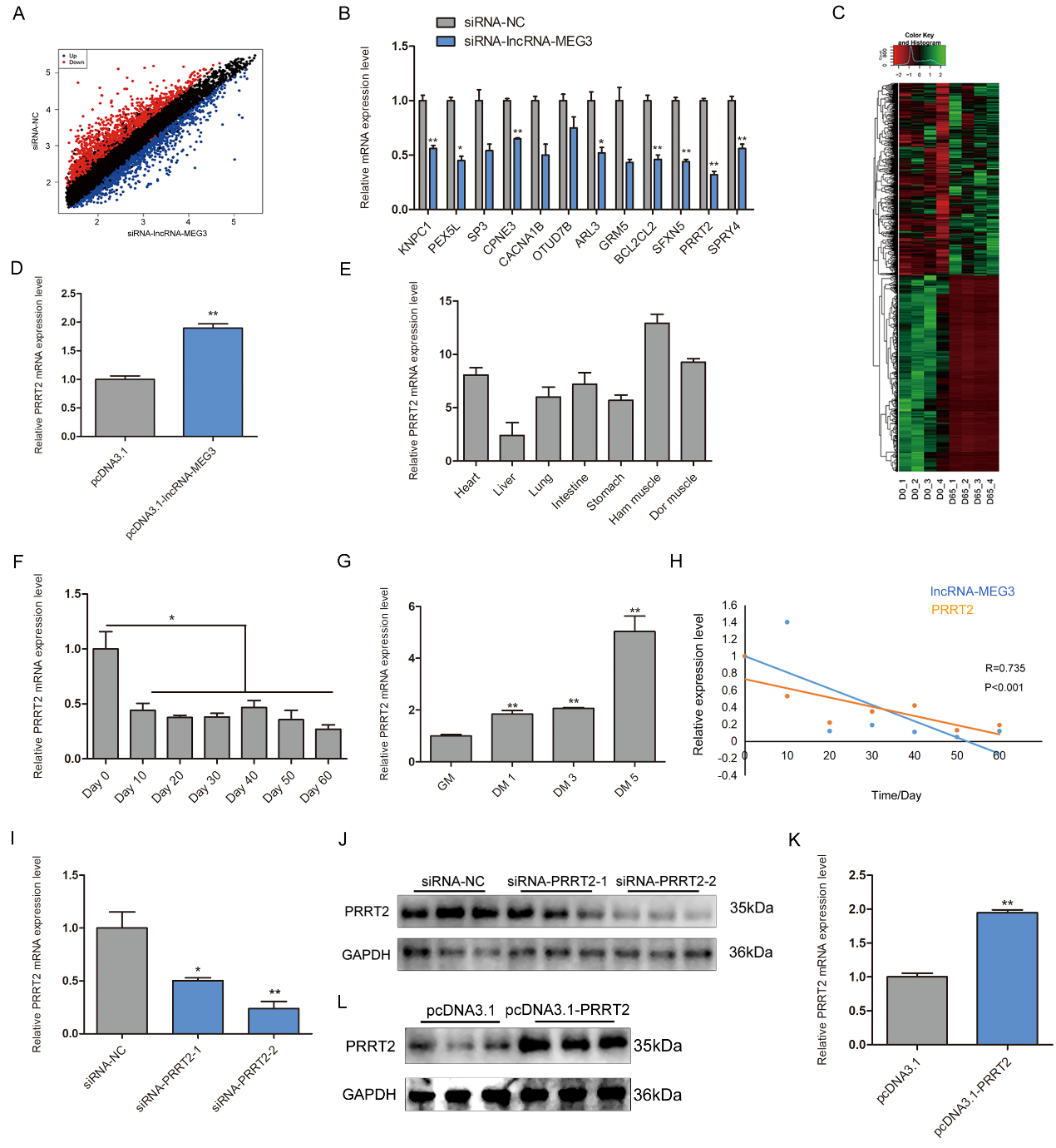


**Figure S9.** Expression patterns of *PRRT2*. **(A)** Scatter plot visualizing differentially expressed mRNAs after the *lncRNA-MEG3* knockdown. **(B)** RT-qPCR was performed to validate the genes randomly selected from the microarray data (*n =*3). **(C)** Heatmap showing 2908 differentially expressed mRNAs between postnatal days 0 and 65. Green indicates low intensity, black indicates medium intensity, and red indicates strong intensity. (D) RT-qPCR analysis of *PRRT2* mRNA expression after *lncRNA-MEG3* overexpression. RT-qPCR analysis of *PRRT2* mRNA expression in seven different tissues of mice at D0 **(E),** in TA muscle from 0 days to 65 days after birth **(F)** and differentiated C2C12 myoblasts **(G)** (*n =* 3). **(H)** Correlation analysis between the spatiotemporal expression of *lncRNA-MEG3* and *PRRT2* during muscle development from D0 to D65. The R-value and the p-value were calculated using the R package function named cor. test. **(I-L)** RT-qPCR and Western blotting analysis of *PRRT2* knockdown and overexpression efficiency (*n =* 3). Data are expressed as mean values ± SEM, and a paired two-tailed Student’s *t*-test was used to analyze the statistical
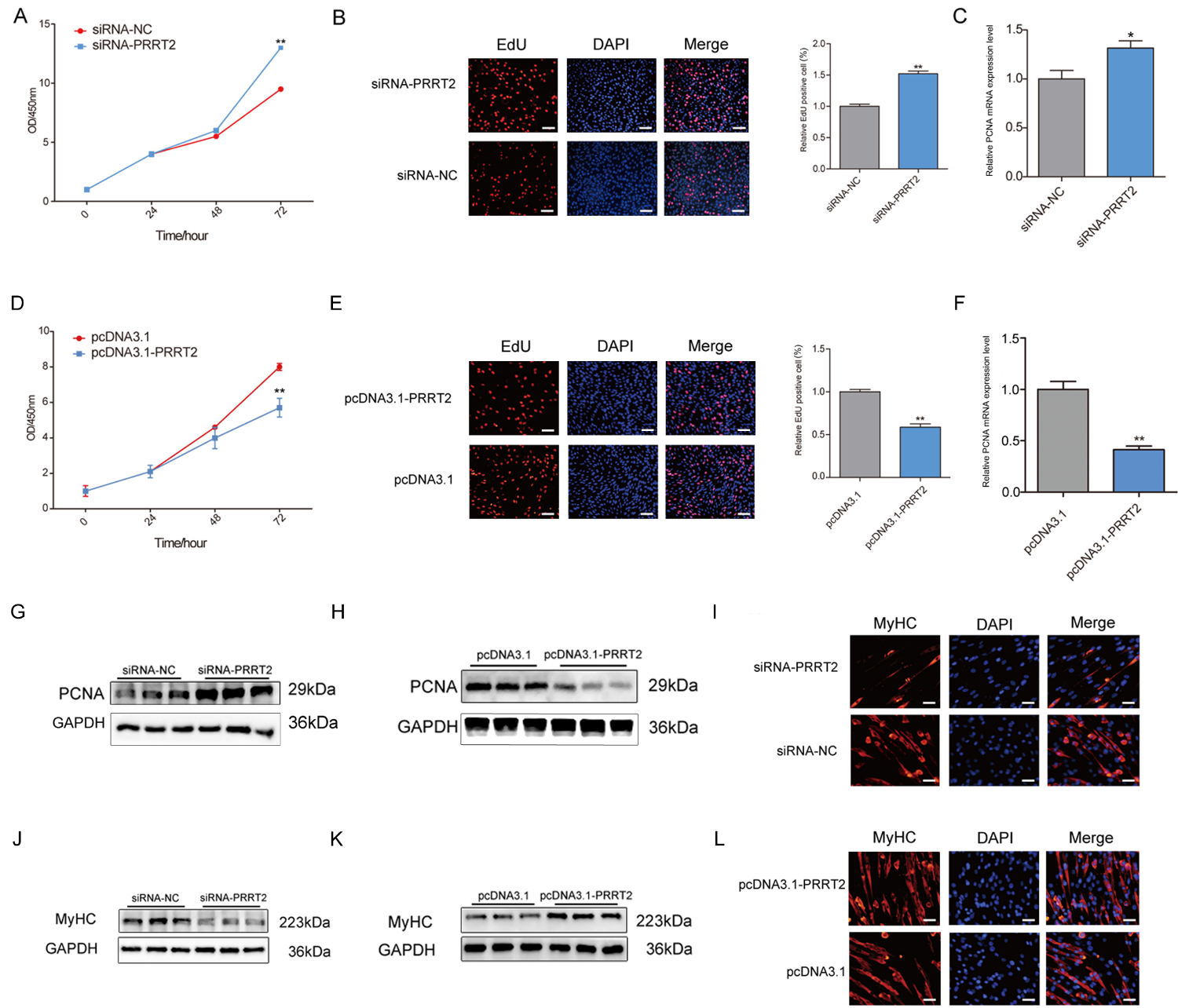
significance between two groups. ** *p* <0.01, and * *p* < 0.05.

**Figure S10.** *PRRT2* inhibited C2C12 myoblasts proliferation and promoted differentiation. **(A-C)** C2C12 myoblasts were transfected with siRNA-NC and siRNA-PRRT2. **(A, B)** Cell proliferation was determined by CCK-8 assay and EdU-staining (*n =* 3). Nuclei were stained with DAPI; scale bar, 50 μm. **(C)** *PCNA* mRNA expression was determined by RT-qPCR in different treatment groups (*n =* 3). **(D-F)** C2C12 myoblasts were transfected with control pcDNA3.1 and pcDNA3.1-PRRT2. **(D, E)** Cell proliferation was determined by CCK-8 assay and EdU-staining in different treatment groups (*n =* 3). Nuclei were stained with DAPI; scale bar, 50 μm. **(F)** *PCNA* mRNA expression was determined by RT-qPCR in different treatment groups (*n =* 3). **(G)** Western blotting analysis of *PCNA* protein expression after *PRRT2* knockdown (*n =* 3). **(H)** Western blotting analysis of *PCNA* protein expression after *PRRT2* overexpression (*n =* 3). **(I)** Immunofluorescence staining analysis of C2C12 differentiation for four days after *PRRT2* knockdown (*n =* 3). Nuclei were stained with DAPI, Scale bar, 20 μm. **(J)** Western blotting analysis of MyHC after *PRRT2* knockdown (*n =* 3). **(K)** Western blotting analysis of MyHC after *PRRT2* overexpression (*n =* 3). **(L)** Immunofluorescence staining analysis of C2C12 differentiation for four days after *PRRT2* overexpression (*n =* 3). Nuclei were stained with DAPI, Scale bar, 20 μm. Data are expressed as mean values ± SEM, and a paired two-tailed Student’s *t*-test was used to analyze the statistical significance between two groups. ∗∗ *p* < 0.01 and ∗ *p* < 0.05.


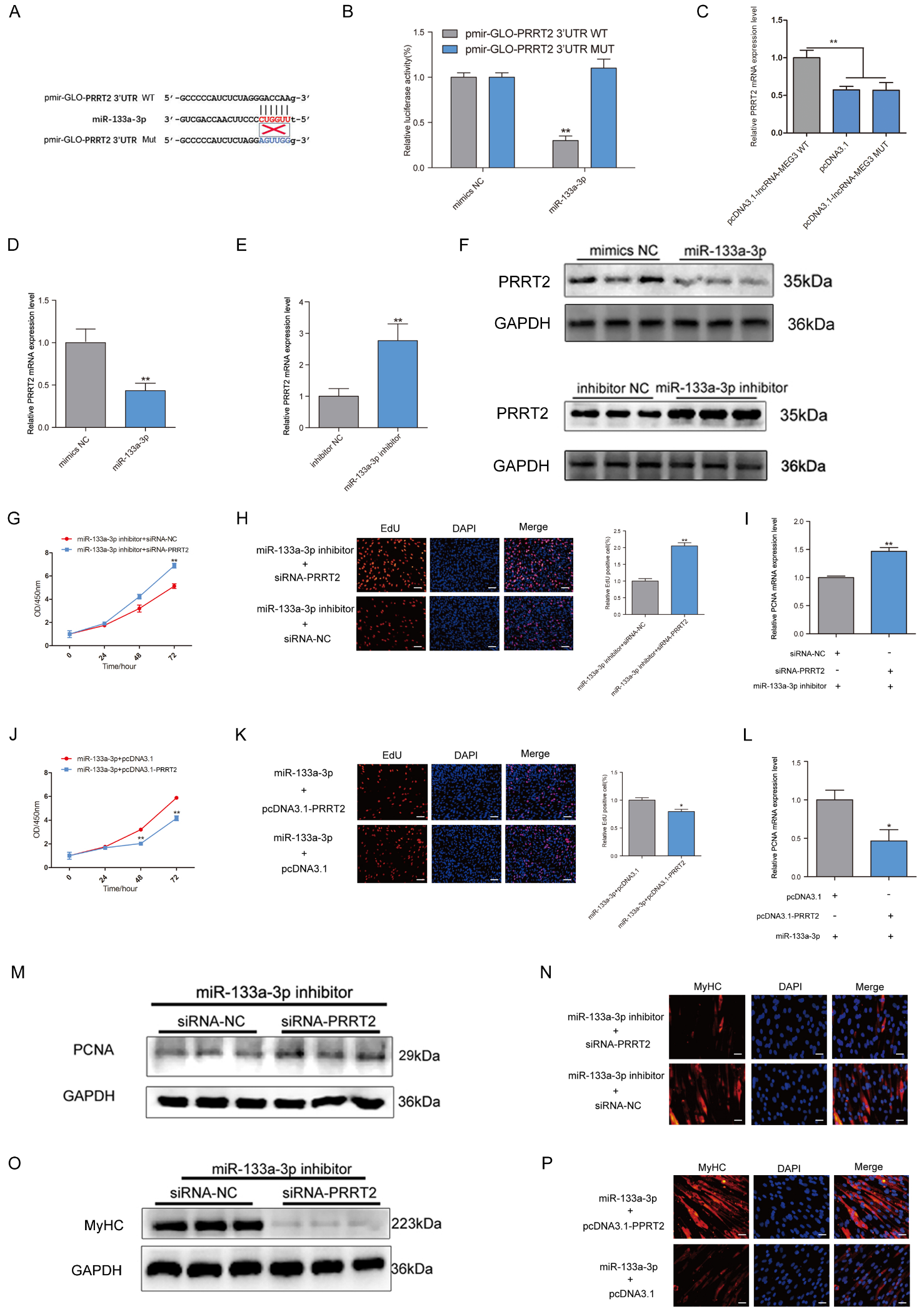


**Figure S11.** MiR-133a-3p targets *PRRT2* to regulate C2C12 myoblasts proliferation and differentiation. **(A)** Construction of pmir-GLO vectors including wild-type (pmir-GLO-PRRT2 WT) and mutant (pmir-GLO-PRRT2 Mut) sequences of *PRRT2 3’UTR*. **(B)** Determination of luciferase activities. miR-133a-3p and mimics NC were co-transfected with the pmir-GLO-PRRT2 WT and pmir-GLO-PRRT2 Mut plasmid into HEK293T cells for 24 h, and the normalized relative luciferase activities ([Renilla](https://www.sciencedirect.com/topics/biochemistry-genetics-and-molecular-biology/renilla)/firefly) were determined (*n* = 3). **(C)** RT-qPCR analysis of *PRRT2* expression in the C2C12 myoblasts treated with pcDNA3.1, pcDNA3.1-lncRNA-MEG3 MUT, or pcDNA3.1-lncRNA-MEG3 WT plasmid (*n =* 3). **(D)** RT-qPCR analysis of *PRRT2* expression after transfection with mimics NC or miR-133a-3p mimics (*n* = 3). **(E)** RT-qPCR analysis of *PRRT2* expression after transfection with inhibitor NC or miR-133a-3p inhibitor (*n* = 3). **(F)** Western blotting analysis of *PRRT2* expression after miR-133a-3p overexpression or knockdown (*n* = 3). **(G-I)** C2C12 myoblasts were transfected with siRNA-PRRT2+miR-133a-3p inhibitor or siRNA-NC+miR-133a-3p inhibitor. **(G, H)** Cell proliferation was determined by CCK-8 assay and EdU-staining in different treatment groups, nuclei were stained with DAPI; scale bar, 50 μm (*n* = 3). **(I)** *PCNA* expression were determined by RT-qPCR in different treatment groups (*n* = 3). **(J-L)** C2C12 myoblast was transfected with pcDNA3.1-PRRT2+miR-133a-3p or pcDNA3.1+miR-133a-3p. **(J)** CCK-8 assay analysis of cell proliferation in different treatment groups (*n* = 3). **(K)** Cell proliferation was determined by EdU-staining in different treatment groups; nuclei were stained with DAPI. Scale bar, 50 μm (*n* = 3). **(L)** *PCNA* mRNA expression was determined by RT-qPCR in different treatment groups (*n* = 3). **(M)** *PCNA* protein expression was determined by Western blotting after C2C12 transfected with siRNA*-PRRT2*+miR-133a-3p inhibitor or siRNA-NC+miR-133a-3p inhibitor. (*n* = 3). **(N)** Immunofluorescence staining analysis of C2C12 differentiated after transfection with siRNA-PRRT2+miR-133a-3p inhibitor or siRNA-NC+miR-133a-3p inhibitor. Nuclei were stained with DAPI; scale bar, 20 μm (*n* = 3). **(O)** Western blotting of *MyHC* after C2C12 transfected with siRNA-PRRT2+miR-133a-3p inhibitor or siRNA-NC+miR-133a-3p inhibitor. (*n* = 3). [C2C12](https://www.sciencedirect.com/topics/biochemistry-genetics-and-molecular-biology/c2c12) myoblasts differentiated in DM for four days **(P)** Immunofluorescence staining analysis of C2C12 differentiation after transfection with pcDNA3.1-PRRT2+miR-133a-3p or pcDNA3.1+miR-133a-3p. Nuclei were stained with DAPI; scale bar, 20 μm (*n* = 3). Data are expressed as mean values ± SEM, and a paired two-tailed Student’s *t*-test was used to analyze the statistical significance between two groups. ∗∗ *p* < 0.01 and ∗ *p* < 0.05.


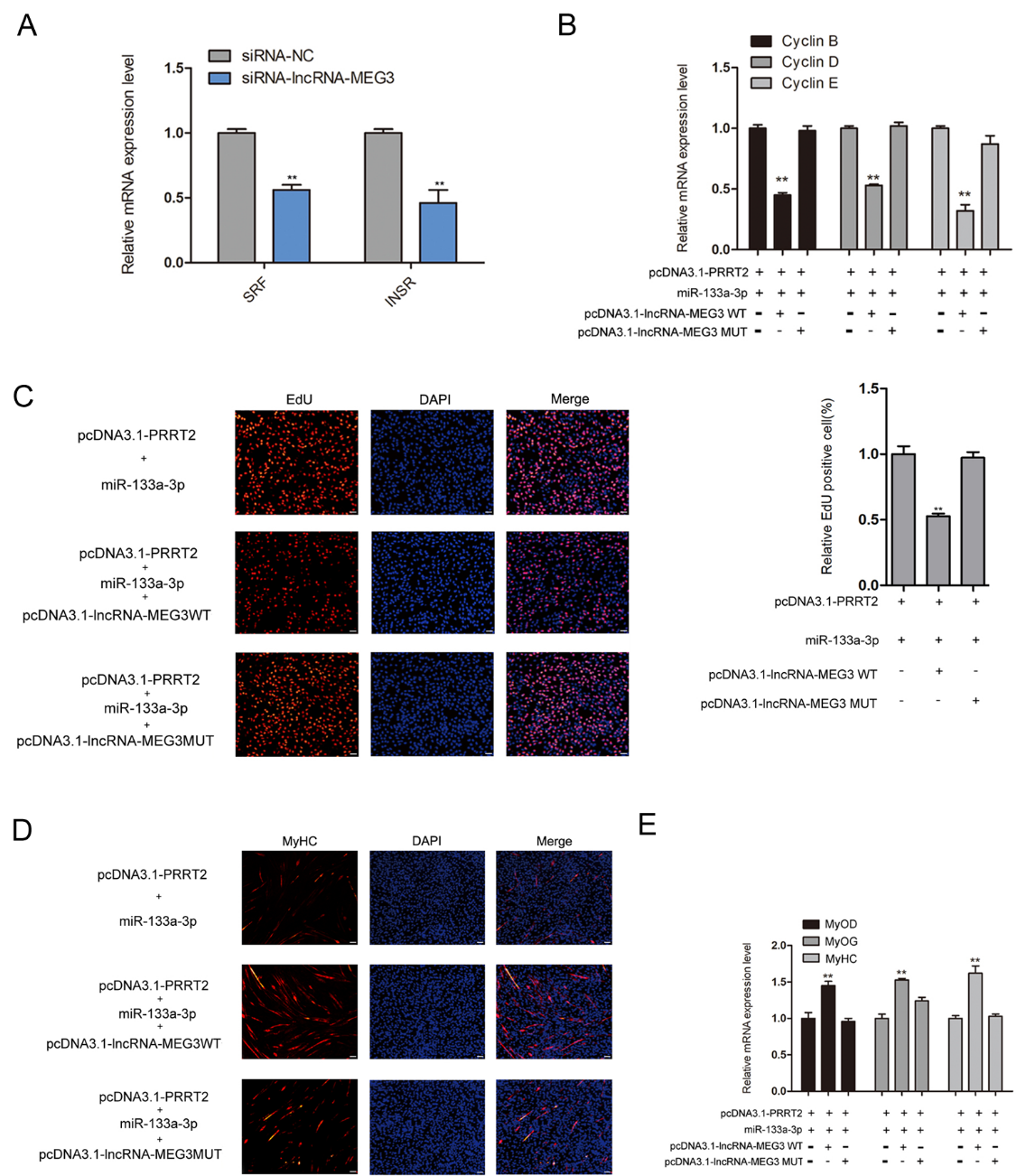


**Figure S12.** *LncRNA-MEG3* controls skeletal muscle differentiation and regeneration via the ceRNA mechanism. **(A)** RT-qPCR analysis of *SRF* and *INSR* mRNA expression level in C2C12 myoblasts after *lncRNA-MEG3* knockdown (*n* = 3). **(B)** RT-qPCR analysis proliferation related marker genes mRNA expression in different treatment groups (*n* = 3). **(C)** EdU-staining analysis for cell proliferation in different treatment groups (*n* = 3). Nuclei were stained with DAPI; scale bar, 100 μm. **(D)** I[mmunofluorescence](http://www.baidu.com/link?url=sFpATC4vsBgFPB6V3HI3xC02ySIY7qu3YnfcBf0BYbTdjYyqV6X0wwwWOempdYP-7gjR13S9wwN2A9cr2OUO5POiTfWmYhDO-R3L3unnPor3EWWRG6nAVty6bRwrRm-b) staining analysis C2C12 myoblasts differentiation in different treatment groups (*n* = 3). Nuclei were stained with DAPI; scale bar, 100 μm. **(E)** RT-qPCR analysis differentiation related marker genes mRNA expression in different treatment groups (*n* =3). Data are expressed as mean values ± SEM, and a paired two-tailed Student’s *t*-test was used to analyze the statistical significance between two groups. ∗∗ *p* < 0.01 and ∗ *p* < 0.05.

**Reference**

1. Oprescu SN, Yue F, Qiu J, Brito LF, Kuang S. Temporal Dynamics and Heterogeneity of Cell Populations during Skeletal Muscle Regeneration. *iScience.* 2020;23(4):100993.

2. Giordani L, He GJ, Negroni E, et al. High-Dimensional Single-Cell Cartography Reveals Novel Skeletal Muscle-Resident Cell Populations. *Mol Cell.* 2019;74(3):609-621.e606.
